# Frailty and mortality: an 18-year follow-up study among Finnish community-dwelling older people

**DOI:** 10.1007/s40520-019-01383-4

**Published:** 2019-10-25

**Authors:** Marika Salminen, Anna Viljanen, Sini Eloranta, Paula Viikari, Maarit Wuorela, Tero Vahlberg, Raimo Isoaho, Sirkka-Liisa Kivelä, Päivi Korhonen, Kerttu Irjala, Minna Löppönen, Laura Viikari

**Affiliations:** 1 City of Turku, Welfare Division, Yliopistonkatu 30, 20101 Turku, Finland; 2 Faculty of Medicine, Department of Clinical Medicine, Unit of Family Medicine, University of Turku, 20014 Turku, Finland; 3 Faculty of Medicine, Department of Clinical Medicine, Unit of Geriatrics, University of Turku, Turku City Hospital, Kunnallissairaalantie 20, 20700 Turku, Finland; 4 Municipality of Lieto Health Care Centre, Hyvättyläntie 7, 21420 Lieto, Finland; 5 Faculty of Medicine, Department of Nursing Science, University of Turku, 20014 Turku, Finland; 6 Turku University of Applied Science, Health and Well-being, Joukahaisenkatu 3, 20520 Turku, Finland; 7 Department of Clinical Medicine, Biostatistics, University of Turku, Turku, Finland; 8 City of Vaasa, Social and Health Care, Ruutikellarintie 4, 65101 Vaasa, Finland; 9 Faculty of Pharmacy, Division of Social Pharmacy, University of Helsinki, 00014 Helsinki, Finland; 10 Faculty of Medicine, Department of Clinical Medicine, Unit of Clinical Chemistry, TYKSLAB, 20521 Turku, Finland; 11 City of Raisio, Social and Health Care for Elderly, Sairaalakatu 5, 21200 Raisio, Finland

**Keywords:** Association, Frailty, Mortality, Older people

## Abstract

**Background:**

There is a lack of agreement about applicable instrument to screen frailty in clinical settings.

**Aims:**

To analyze the association between frailty and mortality in Finnish community-dwelling older people.

**Methods:**

This was a prospective study with 10- and 18-year follow-ups. Frailty was assessed using FRAIL scale (FS) (*n* = 1152), Rockwood’s frailty index (FI) (*n* = 1126), and PRISMA-7 (*n* = 1124). To analyze the association between frailty and mortality, Cox regression model was used.

**Results:**

Prevalence of frailty varied from 2 to 24% based on the index used. In unadjusted models, frailty was associated with higher mortality according to FS (hazard ratio 7.96 [95% confidence interval 5.10–12.41] in 10-year follow-up, and 6.32 [4.17–9.57] in 18-year follow-up) and FI (5.97 [4.13–8.64], and 3.95 [3.16–4.94], respectively) in both follow-ups. Also being pre-frail was associated with higher mortality according to both indexes in both follow-ups (FS 2.19 [1.78–2.69], and 1.69 [1.46–1.96]; FI 1.81[1.25–2.62], and 1.31 [1.07–1.61], respectively). Associations persisted even after adjustments. Also according to PRISMA-7, a binary index (robust or frail), frailty was associated with higher mortality in 10- (4.41 [3.55–5.34]) and 18-year follow-ups (3.78 [3.19–4.49]).

**Discussion:**

Frailty was associated with higher mortality risk according to all three frailty screening instrument used. Simple and fast frailty indexes, FS and PRISMA-7, seemed to be comparable with a multidimensional time-consuming FI in predicting mortality among community-dwelling Finnish older people.

**Conclusions:**

FS and PRISMA-7 are applicable frailty screening instruments in clinical setting among community-dwelling Finnish older people.

**Electronic supplementary material:**

The online version of this article (10.1007/s40520-019-01383-4) contains supplementary material, which is available to authorized users.

## Introduction

Frailty is a problematic clinical syndrome [1]. It is defined as a loss of resources in several domains leading to the inability to respond to physical or psychosocial stress [1–3]. Frailty predicts increased falls, hospitalization, dependence, morbidity, mortality and increase in healthcare costs [1, 2, 4–7]. Prevalence of frailty has varied to some extent between studies, but it has been found to increase with age and to be higher in women than in men [8–13]. Women seem to tolerate frailty better than men, as demonstrated by a lower mortality rate at any given level of frailty or age among women [11, 14]. Anyhow, timely identification of older adults who are frail or at risk of frailty constitutes a cornerstone of geriatric medicine and quality care for the growing elderly population [2, 15–17].

There is no consensus about the key components and assessment of frailty [3, 6, 18]. The ability to predict adverse outcomes is the critical point to determine whether an assessment instrument of frailty is effective or not [18]. FRAIL scale (FS) is judged to be clinically advantageous due to its simple nature and ability to be obtained from data already included in a comprehensive geriatric assessment (CGA) [19, 20]. Also PRISMA-7 is a fast and easily implemented frailty tool in clinical practice [21] and it has been found to have high sensitivity and moderate specificity for identifying frailty among community-dwelling older people [21, 22]. Rockwood’s frailty index (FI), instead, is more comprehensive or prognostic index [23]. FI is well validated and has been applied to multiple datasets [17]. Nonetheless, frailty should be recognized in the clinical setting. According to the Frailty consensus, all persons older than 70 years and all persons with significant weight loss (≥ 5%) due to a chronic disease should be screened for frailty [24]. Screening for frailty helps clinicians to identify and manage the condition early into its progression, facilitate clinical decision making and enable to identify those who need comprehensive geriatric assessment (CGA), followed by targeted interventions to improve quality of life, prevent adverse outcomes, as well as promote an improved allocation of health care resources [21, 25–27].

The aim of this study was to analyze whether frailty, defined with three different frailty tools, FS, PRISMA-7, and FI, was associated with higher mortality risk among Finnish community-dwelling older people during 10- and 18-year follow-ups.

## Materials and methods

### Study design and population

This study is a part of the longitudinal epidemiological study carried out in the municipality of Lieto in southwest Finland [28]. All persons born in or prior to the year 1933 (*N* = 1596) were invited to participate in the baseline examination which was carried out between March 1998 and September 1999. Of those eligible, 63 died before they were examined and 273 refused or did not respond, leaving 1260 (82%) participants, 533 men and 727 women. Subjects living in institutional care (*n* = 65) or in sheltered housing (*n* = 18) or with missing data of frailty indexes were excluded from the analyses.

### Frailty

Frailty was characterized using three commonly used approaches: FRAIL scale (FS) [19], Rockwood’s frailty index (FI) [29, 30], and PRISMA-7 [31, 32]. Data of frailty were gathered using an interview and clinical examination [28] as well as patient records.

The FS, a 5-item self-reported frailty screening tool, includes fatigue, resistance, ambulation, illnesses, and loss of weight components. One point was assigned for each component. Respondents were classified as robust (0 points), pre-frail (1–2 points), or frail (≥ 3 points) according to the total score [19]. We used slightly modified version of FS (Appendix A). In addition, data of illnesses were gathered from patient records instead of self-reporting.

FI consists of at least 30 deficits, such as symptoms, signs, disabilities, and laboratory measurements, which are readily available in survey or clinical data [4, 33]. In this study, FI consisted of 36 items as described in Appendix B. For the level of frailty, three groups were identified using previously described cut points: robust (FI ≤ 0.08), pre-frail, and frail (FI ≥ 0.25) [34].

PRISMA-7 contains seven simple self-reported items to identify frailty: age over 85 years, male gender, health problems which limit activities, support of another person needed, health problems requiring staying at home, social support, and use of a walking aid or a wheelchair. One point is given for every “yes” responses. Respondents with a score of 0–2 are considered to be robust and those with a score of 3 or more are considered to be frail [22, 31]. In our study, three items of the original PRISMA-7 were replaced (Appendix 3).

### Mortality

Data from all participants who died before January 2017 were obtained from the official Finnish Cause of Death Registry using unique personal identification numbers.

### Ethics

The study was conducted according to the guidelines of the Declaration of Helsinki. The Ethics Committee of the Hospital District of Southwest Finland approved the study protocol. Participants provided written informed consent for the study.

### Statistical analyses

At baseline, differences between women and men were tested using the Chi squared test, Fisher’s exact test or two-sample *t* test.

Hazard ratios (HRs) and their 95% confidence intervals for all-cause mortality were calculated using Cox proportional hazard models. Proportional hazards assumption was tested using Martingale residuals. The follow-up periods were calculated from baseline measurements to the end of the follow period of 10 and 18 years or to the death of the person. Firstly, unadjusted Cox regression analyses were conducted for all three frailty indexes. Secondly, Cox regression analyses for FS and FI indexes were adjusted for age and gender which were items of PRISMA-7. The interaction between gender and frailty indexes was included in Cox regression models. *P* values less than 0.05 were considered statistically significant. All statistical analyses were performed using SAS System for Windows, version 9.4 (SAS Institute Inc., Cary, NC, USA).

## Results

### Baseline characteristics

The mean age of the participants was 72.7 (SD 6.2, range 64.0–97.0) years, and 57% were female. More detailed baseline characteristics of 1152 participants are shown in Table 1. Two percent of the participants were identified as frail with FS, 24% with FI, and 17% with PRISMA-7. Frailty (both pre-frailty and frailty) was more common among women than men according to FS and FI; according to PRISMA-7, more men than women were frail. Altogether 1083 participants had frailty assessed with all three indexes, and only one-fifth (20%) was categorized as frail or robust identically according to all three indexes (2% frail, 18% robust). Table 2 shows overlaps of FS, FI, and PRISMA-7.

**Table 1:** Baseline characteristics of study participants (*n* = 1152)

	*n* (%)
Age, mean (SD), range	72.7 (6.2), 64.0–97.0
Age	
64–74	770 (67)
75–84	319 (28)
≥ 85	63 (5)
Female	657 (57)
Living circumstances	
Living with someone	806 (70)
Living alone	346 (30)
Education	
More than basic^a^ or basic	111 (10)
Less than basic	1041 (90)
MMSE	
27–30	879 (76)
< 26	273 (24)
Body mass index, kg/m^2^	
< 20	39 (3)
20–24.9	314 (27)
25–29.9	515 (45)
30–34.9	217 (19)
≥ 35	64 (6)
Smoking	
Non-smokers	751 (66)
Ex-smokers	293 (26)
Current smokers	93 (8)
Frequency of recreational exercising during the previous year	
≥ 3 times a week	611 (55)
Once or twice a week	218 (19)
Less than once a week	290 (26)
Number of prescribed medicines	
< 5	880 (76)
5–7	200 (17)
8–9	51 (4)
≥ 10	21 (2)
Cardiovascular disease	600 (52)
Self-rated health	
Good	473 (41)
Moderate	523 (45)
Poor	156 (14)

^a^Six years of elementary school

**Table 2:** Overlaps of FRAIL scale, frailty index and PRISMA-7 in Finnish community-dwelling older people

	Frailty index (*n* = 1118)				PRISMA-7 (*n* = 1110)		
	Robust	Pre-frail	Frail	*P* value	Robust	Frail	*P* value
FRAIL scale							
Robust	199 (92)	468 (74)	74 (28)	< 0.001	684 (74)	62 (33)	< 0.001
Pre-frail	18 (8)	168 (26)	166 (63)		234 (25)	111 (58)	
Frail	0 (0)	0 (0)	25 (9)		2 (0)	17 (9)	
Frailty index					(*n* = 1090)		
Robust					212 (24)	4 (2)	< 0.001
Pre-frail					583 (65)	50 (27)	
Frail					107 (12)	134 (71)	

### Cox models for frailty and mortality

**Fig. 1:**
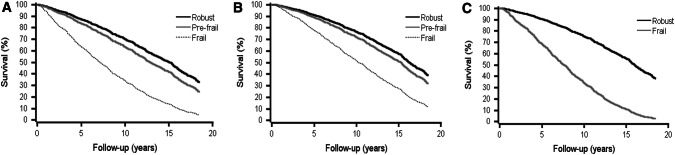
Age- and sex-adjusted survival curves by FRAIL scale (**a**) and frailty index (**b**) and unadjusted survival curves by PRISMA-7 (**c**). The median follow-ups were 14.2 (**a**), 14.3 (**b**), and 14.5(**c**) years

Altogether, 382 (33%) and 776 (67%) subjects deceased during the 10- and 18-year follow-ups, respectively. In unadjusted Cox regression models, both being frail and pre-frail were significantly associated with higher mortality according to FS and FI scales during the 10- and 18-year follow-ups (Table 3). After adjustments for age and gender, these associations remained significant in both follow-ups (Table 4). Also according to binary (robust or frail) PRISMA-7, frailty predicted higher mortality risk. Figure 1 shows age- and gender-adjusted Kaplan–Meier survival curves by FS and FI and unadjusted Kaplan–Meier survival curves by PRISMA-7. The association of frailty, defined by any of the three indexes, and mortality did not significantly differ between men and women either in 10- or 18-year follow-up.

**Table 3:** Unadjusted hazard ratios (HR) and their 95% confidence intervals (CI) (in parentheses) of FRAIL scale, frailty index and PRISMA-7 for all-cause mortality during the 10- and 18-year follow-up

		10-year follow-up		18-year follow-up	
	*n* (%)	HR (95% CI)	*P* value	HR (95% CI)	*P* value
FRAIL scale (*n* = 1152)					
Robust	763 (66)	1		1	
Pre-frail	364 (32)	2.19 (1.78–2.69)	< 0.001	1.69 (1.46–1.96)	< 0.001
Frail	25 (2)	7.96 (5.10–12.41)	< 0.001	6.32 (4.17–9.57)	< 0.001
Frailty index (*n *= 1126)					
Robust	217 (19)	1		1	
Pre-frail	642 (57)	1.81 (1.25–2.62)	0.002	1.31 (1.07–1.61)	0.011
Frail	267 (24)	5.97 (4.13–8.64)	< 0.001	3.95 (3.16–4.94)	< 0.001
PRISMA-7 (*n* = 1124)					
Robust	928 (83)	1		1	
Frail	196 (17)	4.41 (3.55–5.48)	< 0.001	3.78 (3.19–4.49)	< 0.001

**Table 4:** Age- and gender-adjusted hazard ratios (HR) and their 95% confidence intervals (CI) (in parentheses) of FRAIL scale and frailty index for all-cause mortality during the 10- and 18-year follow-up

		10-year follow-up		18-year follow-up	
	*n* (%)	HR (95% CI)	*P* value	HR (95% CI)	*P* value
FRAIL scale (*n* = 1152)					
Robust	763 (66)	1		1	
Pre-frail	364 (32)	1.69 (1.36–2.10)	< 0.001	1.35 (1.15–1.57)	< 0.001
Frail	25 (2)	4.91 (3.10–7.80)	< 0.001	3.92 (2.55–6.01)	< 0.001
Frailty index (*n* = 1126)					
Robust	217 (19)	1		1	
Pre-frail	642 (57)	1.75 (1.21–2.54)	0.003	1.27 (1.03–1.56)	0.026
Frail	267 (24)	4.05 (2.75–5.97)	< 0.001	2.85 (2.25–3.63)	< 0.001

## Discussion

In our study, the prevalence rates of frailty varied from 2% (according to FS) to 24% (according to FI) based on the index used. It is possible that the modified version of FS, used in our study, may have underestimated frailty. In addition, FS is originally designed to be a short screening instrument; FI, instead, is a comprehensive, multidimensional, and more prognostic frailty tool [23].

Both being frail and pre-frail were significantly associated with higher mortality during 10- and 18-year follow-ups both in unadjusted and adjusted models. Also earlier studies with follow-up periods from 2 to 12 years have shown that frailty assessed using FS [20, 35] or FI [30, 33, 36, 37] was a strong predictor of mortality among 65-year-old or older community-dwelling population.

The current study also supports results of the earlier studies showing that frailty indexes differ substantially in how they classified participants as frail [38–41]. However, frailty can potentially be prevented or treated with physical exercise, supplementations, cognitive training and combined treatment, vitamin D, and reduction of polypharmacy [24, 42, 43]. Due to this, the next step is to find out which frail or pre-frail persons can benefit from interventions [41].

Frailty (pre-frailty and frailty) was more common among women than men according to FS and FI; according to PRISMA-7, more men than women were frail. This was probably because according to PRISMA-7 one risk point is given from male gender. However, the association between frailty, defined by any of the three indexes, and mortality did not significantly differ between genders. In another Finnish study [14], frailty was strongly associated with higher mortality, especially among women. Yet, the association between worsening frailty status and mortality risk was more prominent among men. In that population-based study, different frailty assessment was used, participants were older than in our study, and 10% of the participants were institutionalized [14, 44]. Also according to a meta-analysis, in every age group, women had higher FI scores than men but lower mortality rate at any given level of frailty or age suggesting that women tolerated frailty better than men [11].

Three different frailty indexes used in this study are designed for slightly different purposes; FS and PRISMA-7 are designed to be screening instruments and FI to be more comprehensive or prognostic index which characterizes the whole health of an individual [23]. FS is judged to be clinically advantageous due to its simple nature and ability to be obtained from data already included in a patient CGA [19, 20]. Also PRISMA-7 is a fast and easily implemented frailty tool in clinical practice [21] and it has been found to have high sensitivity and moderate specificity for identifying frailty among community-dwelling older people [21, 22]. From five simple instruments to identify frailty in primary care setting, the PRISMA-7 questionnaire achieved the best accuracy and agreement [45]. However, it may over-screen for frailty [22]. The FI, instead, consists of at least 30 deficits, such as symptoms, signs, disabilities, and laboratory measurements, which are readily available in survey or clinical data. This approach does not specify which deficits, or which combinations of deficits, must be present for someone to be considered frail. Also different number of variables can be used [4, 33]. FI is well validated and has been applied to multiple datasets [17]. According to a systematic review [21], specificity of FI was generally high, but sensitivity was low, meaning that it may not identify people who might actually be frail and thereby could miss potentially critical opportunities for treating or supporting these people. In clinical practice, it can be time consuming to calculate FI score [40, 46]. However, implementation of an electronic FI (eFI) that is automatically populated from routine collected data contained within the electronic patient records could represent a major advance in the care of older people with frailty or with a risk of frailty [47].

The strengths of our study are a large sample size and a long follow-up period enabling broad generalizability to the community-dwelling older population. To extend generalizability, we used three validated, commonly used, unweighted frailty indexes [48]. Our analyses also have limitations. We used modified versions of both FS and PRISMA-7 indexes. This may have had impact on frailty classification and predictive validity for mortality [17, 49]. In addition, interpretation of our results (direct comparisons between indexes used) should be made with caution, because scales are designed for different purposes, as described earlier.

Although three frailty indexes captured different groups of individuals, both being frail and pre-frail predicted higher mortality risk according to all three indexes in Finnish community-dwelling older people. Simple and fast frailty indexes, FS and PRISMA-7, seem to be comparable with a multidimensional time-consuming FI in predicting mortality among community-dwelling older people. Therefore, we suggest that FS and/or PRISMA-7 is used for screening older people with frailty of risk of frailty in busy clinical settings. FI, instead, could be used for having a more comprehensive picture of the whole health of older individuals.

## Electronic supplementary material

Below is the link to the electronic supplementary material.

Supplementary material 1 (DOCX 23 kb)

Supplementary material 2 (DOCX 26 kb)

Supplementary material 3 (DOCX 23 kb)
