# Supplementary material for: Frailty and mortality: an 18-year follow-up study among Finnish community-dwelling older people
Source: Aging Clin Exp Res. 2019 Oct 25;32(10):2013–9. doi: 10.1007/s40520-019-01383-4 (PMC7532963; doi:10.1007/s40520-019-01383-4)
Supplement: Supplementary file 1 — Supplementary material 1 (DOCX 23 kb) [file 40520_2019_1383_MOESM1_ESM.docx]

Appendix 1. Modified Frail scale in total population and by gender

| Frail scale items | Total population  (n = 1152)  n (%) | Women  (n = 657)  n (%) | Men  (n = 495)  n (%) | P-value^a^ |
| --- | --- | --- | --- | --- |
| Getting tired for no reason | 259 (22) | 164 (25) | 95 (19) | 0.023 |
| Inability to climb stairs | 69 (6) | 51 (8) | 18 (4) | 0.004 |
| Inability to walk 400 m | 87 (8) | 57 (9) | 30 (6) | 0.115 |
| At least 5 illnesses^b^ | 2 (0) | 1 (0) | 1 (0) | 1.00 |
| Losing weight some of the time–most of the time^c^ | 115 (10) | 67 (10) | 48 (10) | 1.00 |
|  |  |  |  |  |
| Frail scale |  |  |  | 0.025 |
| Robust (0 points) | 763 (66) | 414 (63) | 349 (71) |  |
| Pre-frail (1–2 points) | 364 (32) | 226 (34) | 138 (28) |  |
| Frail (3–5 points) | 25 (2) | 17 (3) | 8 (2) |  |

^a^P-value for the differences between genders

^b^High blood pressure, diabetes, cancer, chronic lung disease, myocardial infarction, congestive heart failure, angina, asthma, arthritis, stroke, and kidney disease.

^c^Original item in Frail scale is as follows: percent weight change ([weight 1 year ago - current weight]/weight 1 year ago) * 100. Percent weight > 5 (representing a 5% loss of weight) is scored as 1 and < 5 as 0.
