# Supplementary material for: Frailty and mortality: an 18-year follow-up study among Finnish community-dwelling older people
Source: Aging Clin Exp Res. 2019 Oct 25;32(10):2013–9. doi: 10.1007/s40520-019-01383-4 (PMC7532963; doi:10.1007/s40520-019-01383-4)
Supplement: Supplementary file 2 — Supplementary material 2 (DOCX 26 kb) [file 40520_2019_1383_MOESM2_ESM.docx]

Appendix 2. Frailty Index in total population and by gender

| Frailty Index items | Total population  (n = 1126)  n (%) | Women  (n = 636)  n (%) | Men  (n = 490)  n (%) | P-value^a^ |
| --- | --- | --- | --- | --- |
| Needs help for toileting | 6 (1) | 3 (0) | 3 (1) | 1.000 |
| Needs help for dressing and undressing | 25 (2) | 9 (1) | 16 (3) | 0.042 |
| Needs help for preparing meals | 111 (10) | 42 (7) | 69 (14) | <0.001 |
| Needs help for house work | 129 (11) | 65 (10) | 64 (13) | 0.157 |
| Needs help for heavy household chores | 358 (32) | 235 (37) | 123 (25) | <0.001 |
| Needs help for personal care | 62 (6) | 39 (6) | 23 (5) | 0.357 |
| Needs help for moving about inside house | 41 (4) | 30 (5) | 11 (2) | 0.036 |
| Arthritis or rheumatism | 78 (7) | 39 (6) | 39 (8) | 0.239 |
| High blood pressure | 397 (35) | 240 (38) | 157 (32) | 0.051 |
| Chronic bronchitis or emphysema | 43 (4) | 13 (2) | 30 (6) | <0.001 |
| Diabetes mellitus | 132 (12) | 69 (11) | 63 (13) | 0.306 |
| Heart disease | 334 (3) | 176 (28) | 158 (32) | 0.100 |
| Cancer | 103 (9) | 61 (10) | 42 (9) | 0.603 |
| Stomach or intestinal ulcers | 63 (6) | 31 (5) | 32 (7) | 0.241 |
| Suffers from the effect of stroke | 43 (4) | 15 (2) | 28 (6) | 0.004 |
| Urinary incontinence | 220 (20) | 193 (31) | 27 (6) | <0.001 |
| Stool incontinence | 5 (0) | 5 (1) | 0 (0) | 0.073 |
| Hip or femoral fracture | 6 (1) | 5 (1) | 1 (0) | 0.241 |
| Shortness of breath | 723 (67) | 418 (68) | 305 (64) | 0.137 |
| Angina pectoris | 520 (46) | 279 (44) | 241 (49) | 0.091 |
| Other medical problems | 648 (58) | 389 (61) | 259 (53) | 0.006 |
| No regular physical exercise | 283 (26) | 171 (28) | 112 (23) | 0.126 |
| Vision problem | 44 (4) | 31 (5) | 13 (3) | 0.063 |
| Hearing problem | 47 (4) | 18 (3) | 29 (6) | 0.015 |
| Feeling hopeless | 183 (16) | 112 (18) | 71 (15) | 0.167 |
| Emotional problem | 80 (71) | 55 (9) | 25 (5) | 0.026 |
| Memory problem | 94 (9) | 45 (7) | 49 (10) | 0.082 |
| Bodily pain | 534 (47) | 321 (50) | 213 (43) | 0.022 |
| Speech problem | 4 (1) | 0 (0) | 4 (2) | 0.127 |
| Resting tremor | 36 (3) | 22 (3) | 14 (3) | 0.612 |
| Five or more medications | 391 (35) | 243 (38) | 148 (30) | 0.006 |
| Difficulties carrying or lifting light loads | 444 (39) | 311 (49) | 133 (27) | <0.001 |
| Mobility problem | 165 (15) | 107 (17) | 58 (12) | 0.022 |
| Limited kind of amount of activity | 85 (8) | 51 (8) | 34 (7) | 0.570 |
| Feeling tired all the time | 34 (3) | 22 (3) | 12 (3) | 0.382 |
| Weight loss | 526 (47) | 293 (46) | 233 (48) | 0.630 |
|  |  |  |  |  |
| Frailty Index |  |  |  | <0.001 |
| Robust (≤0.08) | 217 (19) | 95 (15) | 122 (25) |  |
| Pre-frail (0.09–0.24) | 642 (57) | 369 (58) | 273 (56) |  |
| Frail (≥0.25) | 267 (24) | 172 (27) | 95 (19) |  |

^a^P-value for the differences between genders
