# Supplementary material for: Frailty and mortality: an 18-year follow-up study among Finnish community-dwelling older people
Source: Aging Clin Exp Res. 2019 Oct 25;32(10):2013–9. doi: 10.1007/s40520-019-01383-4 (PMC7532963; doi:10.1007/s40520-019-01383-4)
Supplement: Supplementary file 3 — Supplementary material 3 (DOCX 23 kb) [file 40520_2019_1383_MOESM3_ESM.docx]

Appendix 3. Modified PRISMA-7 in total population and by gender

| PRISMA-7 items | Total population  (n = 1124)  n (%) | Women  (n = 634)  n (%) | Men  (n = 490)  n (%) | P-value^a^ |
| --- | --- | --- | --- | --- |
| Age > 85 years | 40 (4) | 26 (4) | 14 (3) | 0.264 |
| Male | 490 (44) | 0 (0) | 490 (100) |  |
| Rather poor or poor self-rated health^b^ | 145 (13) | 79 (12) | 66 (13) | 0.617 |
| Need for help with taking care of personal hygiene and/or with taking care of finances^c^ | 125 (11) | 80 (13) | 45 (9) | 0.069 |
| Inability to move outdoors^d^ | 37 (3) | 27 (4) | 10 (2) | 0.038 |
| Having someone close to count on in a case of need for help | 1036 (92) | 587 (93) | 449 (92) | 0.555 |
| Use of a stick, walker or wheelchair | 162 (14) | 108 (17) | 54 (11) | 0.004 |
|  |  |  |  |  |
| PRISMA-7 |  |  |  | <0.001 |
| Robust (0–2 points) | 928 (83) | 557 (88) | 371 (76) |  |
| Frail (≥3 points) | 196 (17) | 77 (12) | 119 (24) |  |

^a^P-value for the differences between genders

^b^Original item in PRISMA-7 is as follows: health problems which limit activities

^c^Original item in PRISMA-7 is as follows: support of another person needed

^d^Original item in PRISMA-7 is as follows: health problems requiring staying at home
